# Effects of error-correction of heterozygous next-generation sequencing data

**DOI:** 10.1186/1471-2105-15-S7-S3

**Published:** 2014-05-28

**Authors:** M Stanley Fujimoto, Paul M Bodily, Nozomu Okuda, Mark J Clement, Quinn Snell

**Affiliations:** 1 Department of Computer Science, Brigham Young University, 3361 TMCB PO Box 26576, Provo, UT 84606 USA

## Abstract

**Background:**

Error correction is an important step in increasing the quality of next-generation sequencing data for downstream analysis and use. Polymorphic datasets are a challenge for many bioinformatic software packages that are designed for or assume homozygosity of an input dataset. This assumption ignores the true genomic composition of many organisms that are diploid or polyploid. In this survey, two different error correction packages, Quake and ECHO, are examined to see how they perform on next-generation sequence data from heterozygous genomes.

**Results:**

Quake and ECHO perform well and were able to correct many errors found within the data. However, errors that occur at heterozygous positions had unique trends. Errors at these positions were sometimes corrected incorrectly, introducing errors into the dataset with the possibility of creating a chimeric read. Quake was much less likely to create chimeric reads. Quake's read trimming removed a large portion of the original data and often left reads with few heterozygous markers. ECHO resulted in more chimeric reads and introduced more errors than Quake but preserved heterozygous markers.

Using real *E. coli *sequencing data and their assemblies after error correction, the assembly statistics improved. It was also found that segregating reads by haplotype can improve the quality of an assembly.

**Conclusions:**

These findings suggest that Quake and ECHO both have strengths and weaknesses when applied to heterozygous data. With the increased interest in haplotype specific analysis, new tools that are designed to be haplotype-aware are necessary that do not have the weaknesses of Quake and ECHO.

## Background

The prevalence of next-generation sequencing (NGS) has increased throughput for generating genomic data and our ability to perform genomic analysis. Genomic analyses continue to increase our ability to understand genetic diseases and disorders.

NGS technologies take short fragments of DNA obtained from the genome of an organism of interest. The fragments are then *sequenced*. Sequencing reads is the process of identifying a fragment's constituent nucleotides through chemical processes. A fragment with all bases identified, whether correctly or incorrectly, forms a *read *[1].

These reads are used in several different types of analyses. One example of this is in genome assembly. The generated reads are *assembled *into the source genome through a process of identifying reads that originated from the same regions and *assembling *them by merging them into longer, contiguous sequences called *contigs*. Assembly is complex and difficult due to the short length of sequenced reads and requires volumes of high fidelity data to be accomplished accurately [2].

NGS technologies are, however, imperfect and misidentify some of the nucleotides contained in a DNA fragment as they are being sequenced [1]. Thus, errors are introduced into the reads. These errors are problematic because they introduce false genetic information into a dataset and complicate genome assemblies. Error correction packages have been created that use different techniques in order to locate misidentified bases during sequencing and to correct them to their true sequence [3]. This allows for recovery of data that could otherwise confound a genome assembler. While error correction software is intended to correct errors, it can also introduce errors into a readset through mis-correction.

Genome assembly becomes even more complicated in the presence of *heterozygosity*. Many organisms have a ploidy greater than one, including humans. Diploid and polyploid organisms inherit genetic information from multiple sources (e.g., a maternal and paternal source). One set of genetic variation inherited from a parent is called a *haplotype*. When sequencing and assembling the genome of an organism, haplotype conservation is important for preserving and understanding the biological reality of an organism's genome. Despite lower heterozygosity rates in humans compared to some organisms [4], proper identification of polymorphism remains fundamental to genotype-phenotype analyses.

Many current genome assemblers do not maintain segregated haplotypes [5]. Conservation of separate haplotypes during genome assembly allows for better understanding of complex diseases and phenotypes that have not been associated with single variations. Haplotype-aware analyses allow for more powerful understanding of haplotypes and phenotypes. They also enable better protein structure prediction [6]. Studies of the effects of heterozygosity on error correction performance are lacking.

In this study, two different error correction techniques are comparatively analyzed by examining their effects on next-generation sequencing data from a heterozygous genome. These two packages are Quake and ECHO.

### Quake

Quake is a *k*-spectrum based approach to base-call error correction. Quake analyzes the *k*-mer coverage distribution. Then, a cut-off is determined from the distribution that identify trusted and untrusted *k*-mers. Trusted *k-mers *are used to identify errors in the readset. A set of possible corrections is made and searched to find the correction with the maximum likelihood of making all *k-mers *trusted. In addition to correcting miscalled bases, Quake also trims reads. *Trimming *is the process of removing the ends of reads to remove low quality bases. Quake requires that the value of *k *be specified at runtime. A formula is given by the creators of Quake to calculate *k *based on genome size [7].

### ECHO

ECHO utilizes a multiple sequence alignment (MSA) approach in order to perform base-call error correction. Based on a probabilistic model, ECHO first clusters reads from the same region of the source genome then corrects the reads. ECHO does not require input of genome size or any other input parameters to be run. The authors state that "[ECHO] explicitly models heterozygosity in diploid genomes and provides a reference-free method for detecting bases that originated from heterozygous sites" [8].

## Results

### Synthetic datasets

#### Quake

**Figure 1:**
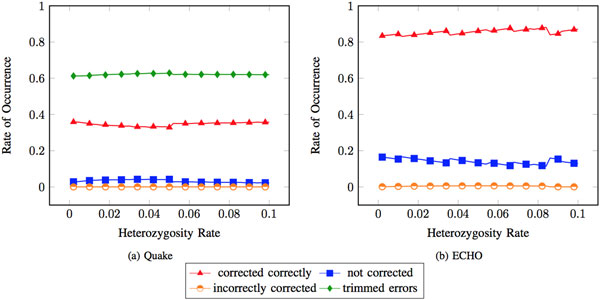
**Errors at heterozygous and homozygous positions (all errors) as treated by Quake and ECHO**. Rate of occurrence is defined as how often an error is treated in a specified way out of all heterozygous and homozygous errors. The haploid genome size was used when running Quake.

Both haploid and diploid genome sizes of the genome were used when calculating the appropriate *k *for Quake. The results from using the haploid and diploid genome sizes had nearly identical results. Quake's general performance does not appear to change much as heterozygosity increases. Quake's performance on both heterozygous and homozygous errors considered together does not appear to change much as heterozygosity increases (see Figure 1).

**Figure 2:**
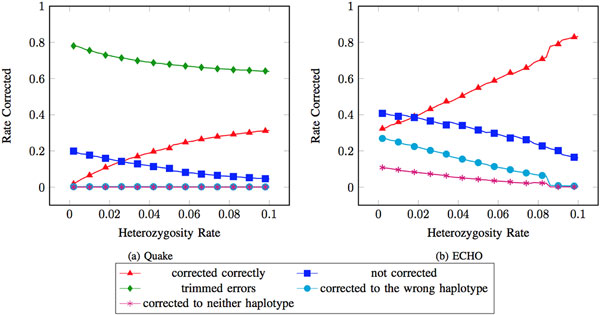
**Errors at only heterozygous positions as treated by Quake and ECHO**. The two error correction programs' performance on errors at heterozygous positions when given the heterozygous dataset at ≈ 3.7% error rate. Rate corrected is defined as the number of corrections made at erroneous heterozygous bases out of all erroneous heterozygous bases in the dataset. The haploid genome size was used when running Quake.

When correcting the datasets with all reads for a particular error and heterozygosity rate combined (*heterozygous dataset*) and using the diploid and the haploid genome sizes as parameters, the error-corrected reads showed several of the same general trends for the first three error rates for errors at heterozygous positions. The rate of errors at heterozygous locations that were corrected correctly increased. The rate of errors at heterozygous locations that were not corrected decreased. The rate of errors at heterozygous locations corrected to the wrong haplotype was low (near 0) and varied little. The rate of errors at heterozygous locations corrected to neither of the haplotypes was low (near 0) and varied little. Finally, the rate of heterozygous locations that had no error corrected to the other haplotype or to neither haplotype was low and varied little (see Figure 2).

A comparison of the first three error rates showed two variations. First, the rate of errors at heterozygous locations corrected to the wrong haplotype increased slightly as the error rate increased. Second, the rate of non-error heterozygous locations corrected to the wrong haplotype was slightly increased with increased error rates.

When correcting the datasets split by haplotype (*homozygous datasets*), several general trends hold for the first three error rates at all levels of heterozygosity. The rate of errors at heterozygous locations corrected correctly was high. The rate of errors at heterozygous locations not corrected was low. The rate of errors at heterozygous locations corrected to the wrong haplotype was low. Lastly, the rate of errors at heterozygous locations corrected to neither of the haplotypes was low.

#### ECHO

ECHO differs from Quake because it does not require the user to specify parameters at runtime. Therefore, ECHO was run once on the heterozygous datasets and again on the homozygous datasets with no variation of parameters. The accuracy of ECHO was similar to Quake as little variation in performance when observing all errors (see Figure 1) was found. The rate of errors at heterozygous locations that were corrected correctly increased. The rate of errors at heterozygous locations corrected to the wrong haplotype decreased. The rate of errors at heterozygous locations that were not corrected decreased. Observed variations between the first three error rates show that the rate of non-error heterozygous locations corrected to the other haplotype decreases as the error rate increases (see Figure 2).

ECHO's correction of the homozygous data for the first three error rates produced trends similar to Quake's. The rate of errors at heterozygous locations corrected correctly was high. The rate of errors at heterozygous locations was low. The rate of errors at heterozygous locations corrected to the wrong haplotype or to neither haplotype was low.

### Real sequencing data

#### Quake

Assemblies using Quake-corrected reads were superior to the uncorrected assembly (see Table 1). The corrected assembly had a decreased number of contigs, larger N50, and an increased largest contig length. The assembly using Quake-corrected reads where the raw reads were segregated by strain during correction performed better than the assembly when all reads were corrected together. N50 nearly doubled when comparing the assembly of reads segregated by strain during correction to the assembly with reads together during correction. The length of the largest contig also increased significantly.

**Table 1:** The SOAPdenovo2 *E. coli *assembly.

Correction Algorithm	How Corrected	Contigs	N50	Largest Contig
	Raw reads	486825	100	7110
Quake	Corrected together	25642	661	28841
Quake	Corrected separately	18153	1143	36891
ECHO	Corrected together	392668	100	10094
ECHO	Corrected separately	348885	100	9563

There were five different assemblies. The assembly of raw reads involved no correction. For both Quake and ECHO, the reads were corrected separately by strain and corrected together with reads from both strains present then assembled. The number of contigs, the N50 size and the length of the largest contig of each assembly is shown.

#### ECHO

Assemblies using ECHO-corrected reads made improvements to the uncorrected assembly (see Table 1). The improvements were not as drastic as those made by Quake. The N50 size remained at 100 base pairs for both assemblies after using ECHO. The largest contig created by both assemblies was better than the uncorrected assembly. It was also slightly improved in the assembly where strains were corrected together compared to when strains were corrected separately. The number of contigs also drops when compared to the uncorrected assembly and is additionally improved when the reads were corrected with strains segregated.

## Discussion

### General trends

Both error correction software packages increased their rate of correctly correcting errors at heterozygous locations as the heterozygosity rate increased. This may be the case because as heterozygosity increases, the haplotypes become more unique. As the sequences become more unique, the error correction algorithms are able to treat the homologs as unique sequences. As can be seen, reads from an organism with high heterozygosity were error corrected better than reads from an organism with low heterozygosity. However, many interesting organisms, such as humans, have low heterozygosity [9].

### Quake

**Figure 3:**
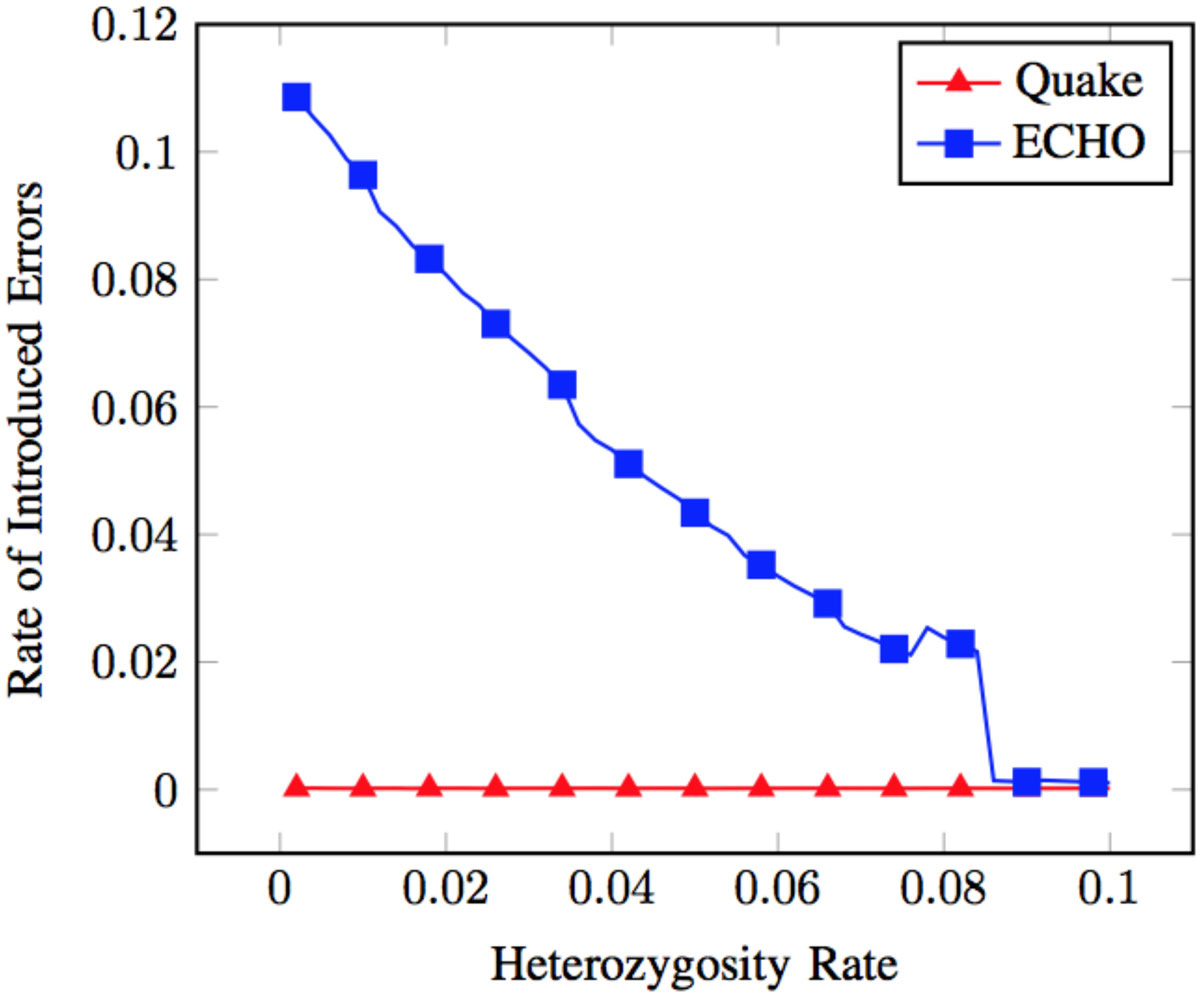
**Introduced errors at non-error heterozygous positions**. Showing heterozygous dataset with ≈ 3.7% error rate where errors were introduced at non-error positions. Introduced errors consisted of non-error bases at heterozygous positions that were corrected to the wrong or neither haplotype. Rate of introduced errors is defined as the number of mis-corrections at non-error heterozygous positions out of all the non-error heterozygous positions in the dataset.

Quake differed from ECHO by its conservative nature when choosing whether or not to error correct. This trend manifested itself in the high rate of errors at heterozygous positions that Quake did not correct when heterozygosity was low. At lower heterozygosity rates, the variations between homologs were sparse. There were, therefore, fewer surrounding heterozygous markers to indicate a proper correction for an error. Quake was less certain of how to correct a putative error at these low heterozygosity rates and preferred to leave them uncorrected. A benefit of this can also be seen by the lower rate at which errors were introduced into the readset by Quake (see Figure 3). Fewer errors being corrected is undesirable but also results in fewer introduced errors.

**Figure 4:**
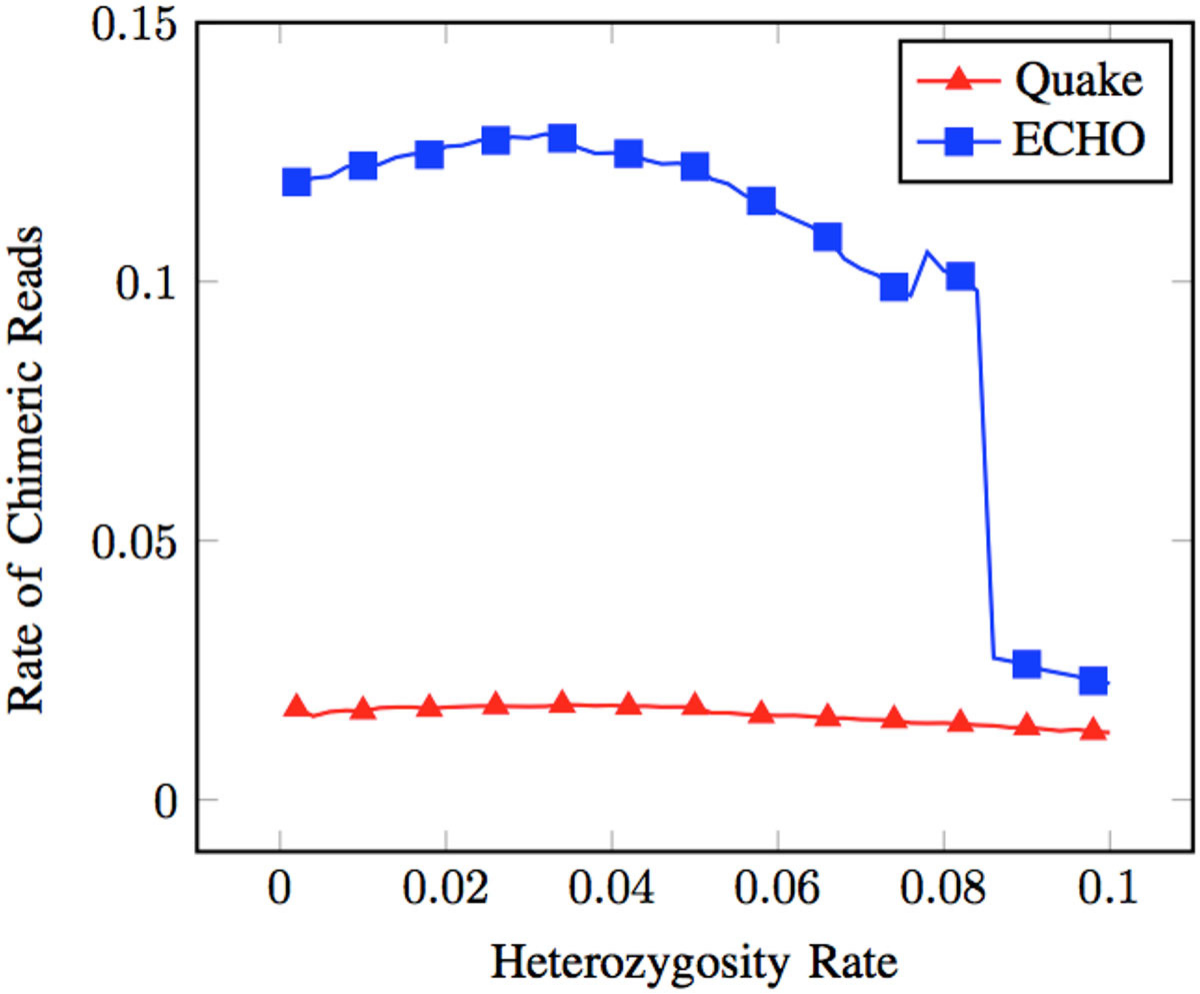
**Chimeric after correction**. Rate chimeric reads is defined as the number of chimeric reads out of all reads that have > 1 heterozygous marker given a heterozygous dataset at ≈ 3.7% error rate.

Quake has a low rate of correcting heterozygous errors to the wrong haplotype. Additionally, it did not introduce errors at non-error heterozygous positions by correcting them to the wrong or neither haplotype (see Figure 3). Additionally, few chimeric reads were present after correction (see Figure 4). The low levels of introduced errors and chimeric reads can be attributed to conservative correcting and trimming of reads.

**Figure 5:**
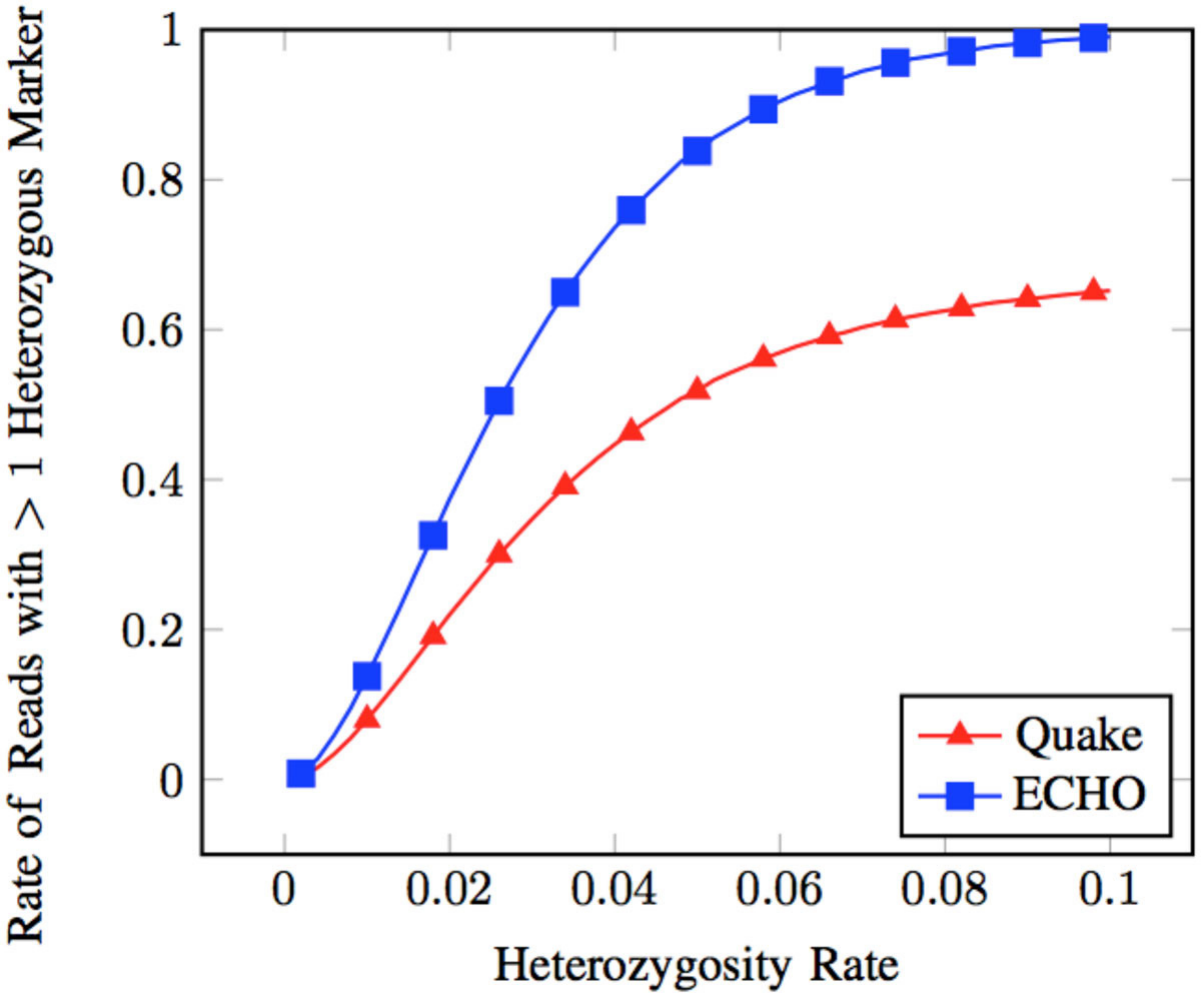
**Reads with > 1 heterozygous marker after correction**. These are reads from the heterozygous dataset at ≈ 3.7% error rate. Rate reads with > 1 heterozygous marker is defined as the number of reads that have > 1 heterozygous marker out of all the reads in the dataset.

Trimming reads may have given Quake an advantage in introducing few errors and creating few chimeric reads. A downside to this, however, can be seen by looking at the proportion of reads that contain > 1 heterozygous marker. As heterozygosity increases, Quake has much fewer reads with > 1 heterozygous marker compared to ECHO (see Figure 5). Reads with > 1 heterozygous marker are essential to haplotype specific genome assembly. Variants contained within the same read can be used to associate them with each other. This association of variants can be used to form a haplotype [10]. Upon further analysis, approximately 60% of all errors in reads that were corrected were removed through trimming (see Figure 1). 40% of these errors were at heterozygous sites when an error rate of approximately 3.7% was used.

### ECHO

One of the strengths of ECHO is that no user input parameters are required in order to run. There is also no trimming. This leaves entire reads intact except for when corrected. The lack of trimming may or may not be a weakness depending on the needs of the user.

ECHO showed itself to be aggressive in terms of error correction. This differentiates it from Quake's conservative tendencies. Aggressive correction proved to be a good feature when presented with homozygous data. However, it was detrimental when heterozygous data was present. At low heterozygosity rates, ECHO corrected errors and non-errors at heterozygous positions to the wrong or neither haplotype approximately 10% of the time in the worst cases at the lowest error rate. ECHO corrected many errors that Quake did not correct at low heterozygosity rates. However, it also introduced more errors into the readset. The effects of introducing errors at heterozygous positions can be seen in the number of chimeric reads after correction.

ECHO produced more chimeric reads than Quake (see Figure 4). As discussed earlier, this feature can be detrimental for downstream use of these corrected reads. The most likely reason for this increase in chimeric reads is the lack of read trimming in ECHO when errors are encountered near the end of a read. This leaves all heterozygous positions present in a read. Having more heterozygous positions present is a key feature for haplotype-aware studies. Compared to Quake, the number of reads that contain > 1 heterozygous base is higher, especially as heterozygosity increases (see Figure 5). Though more reads may be chimeric, the higher number of reads with multiple heterozygous markers may be beneficial.

### Real sequencing data

Using either Quake or ECHO improved the real sequence data genomic assemblies. The overall number of contigs returned after assembly were decreased on all error-corrected assemblies. This is important because with fewer returned contigs, the scaffolding graph is much less complicated. More improvements can be found when using Quake compared to ECHO. It is unclear how much of the improvement that was found after using Quake is attributed to read trimming or due to the correction of errors. The improvements seen in all error-corrected assemblies shows how error correcting can improve downstream analysis. The assembly created from reads corrected by Quake when the reads were separated by strain was significantly better than the assembly produced with mixed reads. A haplotype-aware error correction algorithm should be able to achieve this level of performance.

## Conclusion

Neither Quake nor ECHO performed adequately as an error correction algorithm on heterozygous data. An acceptable error correction algorithm for heterozygous reads would provide results similar to Figure 1 when correcting a heterozygous dataset.

ECHO is a very aggressive corrector. Quake, on the other hand, is much more conservative. Aggressive correction provides the benefit of correcting many of the errors. It also results in the introduction of errors. Many of these errors occur at heterozygous positions. Conservative correction results in more of the original errors persisting through the correction phase but fewer new errors being introduced.

An important distinction between the original errors and introduced errors should be made. Original errors are much less detrimental to downstream analysis than introduced errors. Introduced errors are placed in non-random locations and decrease the ability to compensate for these errors through high coverage.

In addition to correction accuracy, the number of heterozygous markers present after correction should also be considered. Because Quake trims reads, a large portion of heterozygous bases were removed from the datasets. This created fewer errors but left fewer heterozygous markers in the corrected dataset. ECHO, although it had lower accuracy as a result of more introduced errors and mis-corrections of read errors, left all heterozygous positions in the corrected dataset.

Looking at the effects on real sequencing data and their subsequent assemblies was also enlightening and showed effects of error correction on real data. It is shown that both algorithms improve the assembly. The effects of haplotype-aware error correction can particularly be seen in the case of Quake when the reads from each strain were segregated.

There is much improvement that can be made to error correction algorithms to better handle heterozygous datasets. haplotype-aware correction algorithms should be able to enhance genome analysis. Other approaches also include read classification and segregation by haplotype before error correcting. Segregating the data creates datasets that resemble homozygous data. Thus, many current bioinformatic tools could be used on heterozygous datasets that are segregated by haplotype.

## Methods

Heterozygous datasets were created, error correction software packages were applied to the reads, and the corrected reads were analyzed in order to evaluate error correction software performance on heterozygous genomes.

### Data generation

A 4,940,000 base-pair region of chromosome 20 from *Homo sapiens *[Gen-Bank: http://www.ncbi.nlm.nih.gov/nuccore/NT_011387.8] was used as one of the haplotypes. The sequence was then duplicated. HapMaker was used to introduce heterozygosity at 50 different levels (0.2% to 10% at 0.2% intervals) into the duplicate sequence [11]. The heterozygosity introduced duplicate sequence was used as another haplotype. The simulated diploid genome contained reads from the original sequence and the heterozygosity introduced duplicate. Haplotypic differences were limited to single nucleotide base variations. ART was used to generate 75 base pair Illumina single reads at 40x coverage per dataset [12]. For each level of heterozygosity, four datasets at different error rates were created:

1 ≈ 3.7%

2 ≈ 4.6%

3 ≈ 5.8%

4 ≈ 7.3%

This resulted in 200 unique datasets with combinations of different heterozygosity and error rates.

### Using the error correction software

The 200 readsets were corrected separately using Quake and ECHO. For each algorithm, the readsets were corrected once with all of the reads for a particular error and heterozygosity rate present (*heterozygous dataset*) and again with the reads separated by haplotype (*homozygous dataset*). The performance of the different error correction algorithms were then compared for heterozygous and homozygous readsets.

### Analysis

#### Synthetic data

The efficacy of the error correction software packages was measured at the nucleotide and the readset level. This research focusses on base pairs at heterozygous positions. Each base in the readset was examined to determine if the base was modified by the error correction software. A base that was modified by the error correction software was denoted as *corrected*. A heterozygous base pair that is an error or non-error can be handled in several ways:

• *corrected correctly *: a base that is corrected to what is found in the source genome

• *corrected to the wrong haplotype*: a mis-correction, a base at a heterozygous position that is corrected to its homologous pair

• *corrected to neither haplotype*: a mis-correction, a base at a heterozygous position that is corrected to neither of the haplotypes present

• *not corrected *: a base at a heterozygous position is left as found

In addition to the nucleotide level analysis, a readset level analysis was conducted. The number of chimeric reads within a corrected dataset was found. A chimeric read must have > 1 heterozygous marker and have different haplotypes represented at these positions. A non-chimeric read only has one haplotype at all the heterozygous markers it contains. An algorithm with good performance on heterozygous datasets will have few chimeric reads.

This analysis was performed using synthetic genomes based on the human reference genome and readsets where the location of each read and correct nucleotide sequence was known. Unknown sample position and correct sequence of a read makes knowing exactly how the error correction algorithms performed impossible to know for real genomic data. Thus, only single base errors and corrections were analyzed using synthetic data in this study.

#### Real sequencing data

In addition to using synthetic data, an analysis using real data was performed. Genomic assemblies were generated using SOAPdenovo2 [13]. SOAPdenovo2 uses a de Bruijn graph approach to solving the genome assembly problem. The *k-mer *size for these assemblies was set to 31. These assemblies were used to compare the effects of error correction on real data.

Two strains of *Escherichia coli *were selected. Whole shotgun sequence data for the strains [SRA:SRR800579 and SRA:SRR784244] were gathered from the Sequence Read Archive (SRA) hosted by NCBI. These data are 101 base pair Illumina HiSeq 2000 reads at approximately 50x coverage per strain. The reads from these two strains were combined to generate real data that is representative of the sequence data from a diploid organism. The reads were corrected and assembled to see how error correction affected them. This analysis was accomplished by first creating a baseline assembly by using all uncorrected reads from both strains together. Two assembly pipelines were used for both Quake and ECHO:

• Combine reads from both strains, correct the combined file, then assemble

• Keep reads separate by strain, correct the two files separately, combine corrected reads, then assemble

This resulted in four assemblies from corrected reads and one assembly from the uncorrected reads.

The N50 and length of the longest contig were measured for each of the five assemblies. A larger N50 and a longer longest contig are indicators of a higher quality genome assembly. It is expected that the quality of the assemblies at the base pair level will also be higher with corrected readsets.

## Competing interests

The authors declare that they have no competing interests.

## Authors' contributions

MSF developed the experimental design, performed the analyses, and drafted the manuscript. PMB participated in the experimental design and the analyses. NO participated in the experimental design and the analyses. MJC helped experimental design, analyses and oversaw the project. QS helped in the analyses and oversaw the project. All authors read and approved the final manuscript.
